# Correlation of MR-Based Metabolomics and Molecular Profiling in the Tumor Microenvironment of Temozolomide-Treated Orthotopic GL261 Glioblastoma in Mice

**DOI:** 10.3390/ijms242417628

**Published:** 2023-12-18

**Authors:** Kai Zhao, Pilar Calero-Pérez, Miriam H. A. Bopp, Vincent Möschl, Axel Pagenstecher, Marta Mulero-Acevedo, Mario Vázquez, Carlos Barcia, Carles Arús, Christopher Nimsky, Tillmann Rusch, Jörg W. Bartsch, Ana Paula Candiota

**Affiliations:** 1 Department of Neurosurgery, Philipps University Marburg, Baldingerstrasse, 35043 Marburg, Germany; kai.zhao.neurosurgery@gmail.com (K.Z.); bauermi@med.uni-marburg.de (M.H.A.B.); nimsky@med.uni-marburg.de (C.N.); 2 Departament de Bioquímica i Biologia Molecular, Facultat de Biociències, Universitat Autònoma de Barcelona, 08193 Cerdanyola del Vallès, Spain; pilarcalero92@gmail.com (P.C.-P.); marta.mulero@uab.cat (M.M.-A.); mariovp13@gmail.com (M.V.); carlos.barcia@uab.cat (C.B.); carles.arus@uab.cat (C.A.); 3 Centro de Investigación Biomédica en Red: Bioingeniería, Biomateriales y Nanomedicina, 08193 Cerdanyola del Vallès, Spain; 4 Center for Mind, Brain and Behavior (CMBB), Hans-Meerwein-Strasse 6, 35032 Marburg, Germany; pagenste@med.uni-marburg.de; 5 Department of Neuropathology, Philipps University Marburg, Baldingerstrasse, 35043 Marburg, Germany; vincent.moeschl2@uk-gm.de; 6 Department of Neuropathology, Core Facility Mouse Pathology and Electron Microscopy, Philipps-University Marburg, 35037 Marburg, Germany; 7 Institut de Biotecnologia i Biomedicina, Universitat Autònoma de Barcelona, 08193 Cerdanyola del Vallès, Spain; 8 Institut de Neurociències, Facultat de Medicina, Universitat Autònoma de Barcelona, 08193 Cerdanyola del Vallès, Spain; 9 Department of Hematology, Oncology and Immunology, Philipps University Marburg, Baldingerstrasse, 35043 Marburg, Germany; tillmann.rusch@uk-gm.de

**Keywords:** glioblastoma, MR spectroscopic imaging, temozolomide, therapy, metalloproteases, PD-L1, shedding, macrophages

## Abstract

The tumor microenvironment in glioblastoma (GB) is considered to be “cold”, i.e., the fraction of cytotoxic T cells, for instance, is low. Instead, macrophages are the major immune cell population in GB, which stem either from tissue response (resident microglia) or recruitment of macrophages from the periphery, thereby undergoing tumor-dependent “imprinting” mechanisms by which macrophages can adapt a tumor-supportive phenotype. In this regard, it is important to describe the nature of macrophages associated with GB, in particular under therapy conditions using the gold standard chemotherapy drug temozolomide (TMZ). Here, we explored the suitability of combining information from in vivo magnetic resonance spectroscopic (MRS) approaches (metabolomics) with in vitro molecular analyses to assess therapy response and characterize macrophage populations in mouse GB using an isogenic GL261 model. For macrophage profiling, expression levels of matrix metalloproteinases (MMPs) and A disintegrin and metalloproteinases (ADAMs) were determined, since their gene products affect macrophage–tumor cell communication by extensive cleavage of immunomodulatory membrane proteins, such as PD-L1. In tumor mice with an overall therapy response, expression of genes encoding the proteases ADAM8, ADAM10, and ADAM17 was increased and might contribute to the immunosuppressive phenotype of GB and immune cells. In tumors responding to therapy, expression levels of ADAM8 were upregulated by TMZ, and higher levels of PD-L1 were correlated significantly. Using a CRISPR/Cas9 knockout of ADAM8 in GL261 cells, we demonstrated that soluble PD-L1 (sPD-L1) is only generated in the presence of ADAM8. Moreover, primary macrophages from WT and ADAM8-deficient mice showed ADAM8-dependent release of sPD-L1, independent of the macrophage polarization state. Since ADAM8 expression is induced in responding tumors and PD-L1 shedding is likely to decrease the anti-tumor activities of T-cells, we conclude that immunotherapy resistance is caused, at least in part, by the increased presence of proteases, such as ADAM8.

## 1. Introduction

Glioblastoma (GB, formerly termed GBM) is an aggressive and highly malignant type of brain tumor that arises from glial cells in the central nervous system. GB is the most common primary brain tumor in adults, with an incidence of 4 in 100,000 in the population, and is characterized by infiltrative growth patterns, rapid proliferation, and resistance to treatment. Despite advancements in surgery and adjuvant therapy using the standard drug temozolomide (TMZ), the prognosis for GB patients remains poor, with a median survival time of only 15 months [1]. Several factors contribute to this poor prognosis, and it is a well-established concept that GB confers a strong immunosuppressive tumor microenvironment (TME) [2,3,4]. Immunosuppression is worsened by a limited delivery of drugs to the brain due to the blood–brain and blood–tumor barrier and the persistence of glioma stem cells (GSCs), which acquire resistance to therapy, thereby frequently leading to GB recurrence. The TME comprises a complex network of non-cancerous immune cells, extracellular matrix components, signaling molecules, and enzymes that interact with tumor cells [5]. It is critically involved in cancer therapy responses, including GB, and may trigger local changes that can have an impact on non-invasive approaches for tumor follow-up, such as magnetic resonance imaging [6]. MRI as a powerful technique plays a crucial role in the diagnosis, treatment planning, and follow-up of GB, offering structural, functional, and metabolic information (e.g., MR spectroscopy/spectroscopic imaging, MRS/MRSI). In particular, metabolomics is known to produce earlier and more confident information about events related to therapy in preclinical GB models [7]. Thus, local changes during GB therapy triggered by changes in the TME may be spotted by non-invasive approaches, such as MRSI, providing potential clues about efficient tumor elimination by the host immune system, thereby producing potential surrogate biomarkers with oscillatory frequency [8,9,10] matching the immune cycle length. As a major immune cell type in the TME, glioma-associated microglia/macrophages (GAMs) can present various subtypes with a simplified categorization into either pro-inflammatory anti-tumor (M1) or pro-tumor (M2) phenotypes. Metalloproteinases, such as matrix metalloproteases (MMPs) and metalloprotease–disintegrins (ADAMs), as potent mediators of cell-to-cell signaling, are able to modulate the tumor microenvironment and were found to be correlated with GAM phenotypes [11]. Furthermore, carbonic anhydrases (CAs) are metabolic enzymes essential for the regulation of intra- and extracellular pH, i.e., the release of protons in the extracellular compartment, and, as such, they are important for the activation of metalloproteases. Metabolic processes changed in GB lead to acidic conditions that are detrimental to normal cells but have minimal effect on tumor cells, thus supporting tumor formation. As such, the accumulation of acidic metabolites may result from upregulating proton transporters or lactate transporters, such as CAs and monocarboxylate transporters (MCTs) [12]. The dependence on an acidic microenvironment has been the focus of studies on GB therapy resistance. For instance, it was shown that GSCs are resistant to TMZ treatment. We and others have previously shown that a major factor in TMZ resistance in GB stem cells and recurrent tumor tissue is carbonic anhydrase 2 (CA2) [13,14].

One of the current challenges in GB therapy is that most of the clinical and preclinical administration schedules do not consider the cancer immune cycle and may be counterproductive. In previous studies, we have, therefore, used a modified therapeutic protocol that follows an immune-respectful schedule for TMZ administration that greatly improved mouse survival and immune-related parameters [9,15,16], termed the “Immune-enhancing metronomic schedule” (IMS-TMZ, [9]) that we employed throughout the present study. This protocol demonstrated the capability to induce tumor regression and achieve a cure in 50% of the administered cohort. Additionally, the treated subjects displayed immune memory against new tumor inoculations, a phenomenon previously observed in the context of subcutaneous GL261 [17,18]. In previous work [16], significant changes in the TME were described that occurred during response to IMS-TMZ treatment, with regard to the GAM population and aspects correlated with PD-L1 expression. Programmed Death Factor 1 axis (PD-1/PDL-1) could serve as one of the most important immunosuppressive mechanisms in GB. PD-L1 could be a substrate for an extracellular protease of the ADAM family that is highly expressed in tumor cells and macrophages. Given the expression patterns of GB-relevant genes and their relationship to the M1/M2 macrophage populations observed on the spectroscopic and molecular level, we were able to identify correlations between macrophage populations, metabolites, and gene expression patterns that can impact the nature of the TME in GB. 

The overall aim of the present study was to evaluate immune-related changes in the TME of control/untreated and three GB response groups (namely, control mice; IMS-TMZ-treated responding mice; IMS-TMZ-treated relapsing mice; and IMS-TMZ-treated unresponsive mice). Quantitative PCR was used in order to characterize the different subpopulations of GAMs and PD-L1 gene expression, as well as the molecular profile of different protease genes known to be associated with GB progression (ADAM8, ADAM10, ADAM17, MMP9, and MMP14) and genes encoding the most relevant CAs in GB, namely, CA2, CA9, and CA12. Gene expression data were correlated with MRSI data and immunohistochemistry studies to assess the distribution of microglia/macrophages with different phenotypic profiles, using markers for M1- and M2-like macrophages. As an interesting readout of our analysis, the strong correlation of ADAM8 with PD-L1 expression tempted us to analyze PD-L1 release in GB cells and in macrophages in the presence or absence of ADAM8, thereby potentially contributing to a potential mechanism that prevents effective immunotherapy of GB. 

## 2. Results

### Non-Invasive Follow-Up of Tumor Progression in Mice and Inclusion Criteria

In addition to the twenty cases of tumor-implanted mice already described [16], twenty-one additional cases were included to encompass the situations of relapsing and non-responding at different biomarker oscillation time points. Thus, the overall number of individuals in each group and the tumor progression, as well as the euthanasia days, are shown in Table 1.

**Figure 1:**
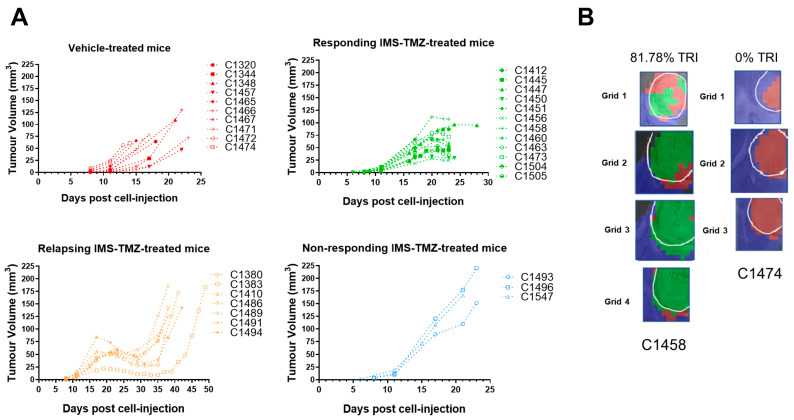
Tumor volume progression in cases used for RT-PCR studies. (**A**) Tumor volume progression for vehicle-treated mice (top, left, red lines) and TMZ-treated mice (top, right, green lines), treated-relapsing mice (bottom, left, orange lines), and treated-unresponsive mice (bottom, right, blue lines). This color coding for the different groups will be used in subsequent figures. TMZ was administered every 6 days, and IMS-TMZ from day 11 p.i. Volumes were calculated from the T_2_-weighted MRI (T_2_w MRI) acquisitions. An example of T_2_w MRI evolution for each group can be found in Supplementary Figure S2. (**B**) Nosological images generated from multi-slice MRSI acquisition, after being analyzed and classified with semi-supervised machine learning approaches (analyzed as in [8,20]), from 2 chosen cases marked in “A”, one IMS-TMZ-treated and responding, and another from control, vehicle-treated groups. Color coding: green, treated responding tumors; red, control/untreated tumors; blue, unaffected brain parenchyma. Mice selected for immunohistochemistry studies followed the same inclusion criteria. See Section 4.3 for the calculation of the tumor responding index (TRI). Tumor volume at the euthanasia time point and euthanasia day can be found in the supplementary information file, as well as an explanation of the nosological image formation. Multi-slice MRSI acquisition had 3 or 4 slices, depending on tumor size and coverage. Top and bottom slices had 10 × 10 matrices, and central slices had 12 × 12 matrices. Pixel sizes in nosological images: 0.5 × 0.5 × 1 mm. Adapted and improved from [16].

The average tumor volume at the therapy starting point (day 11 p.i.) in all groups was within the expected variability registered over many years in our research group. At chosen time points, mice were euthanized by cervical dislocation, brains were removed, and tumors were resected. This timepoint matches the humane endpoint in relapsing and non-responding cases, while vehicle-treated cases and IMS-TMZ-responding cases followed strict inclusion criteria based on tumor volume changes and MRSI-based biomarker appearance (detailed in the Supplementary File). Supplementary Table S1 lists specific details for each sample collected. Figure 1 summarizes the tumor volume evolution in the four groups, as well as examples of nosological images obtained (see Section 4.3 and the Supplementary File for an explanation on how such nosological images were obtained and a definition of the tumor responding index, TRI (Supplementary Figure S1)).

Untreated (control) tumors presented the expected exponential growth. Note that, for this work, the curves shown in Figure 1A for control mice (red lines) do not represent the final humane endpoint, since we wanted to avoid late tumor stages in which excessive necrosis could be found while ensuring that enough samples were available for the required studies. In the case of IMS-TMZ-treated, responding mice, tumors presented the characteristic growth arrest or even volume decrease, which is usually more evident during the second TMZ administration timepoint at day 17 p.i. Samples were obtained at this specific timepoint without allowing further evolution to ensure that we were situated at the point of objective/active response to TMZ therapy. For relapsing cases, IMS-TMZ-treated tumors presented transient growth arrest followed by exponential regrowth situated around day 30–35 p.i., in which tumor relapsing became evident. Finally, in IMS-TMZ-treated, unresponsive cases, tumors did not show any sign of growth arrest or decrease, behaving mostly as untreated cases. It is worth noting that these cases have a lower number of representative cases since it is unusual that GL261 GB-bearing mice do not present even transient responses to the IMS-TMZ therapeutic protocol. 

**Figure 2:**
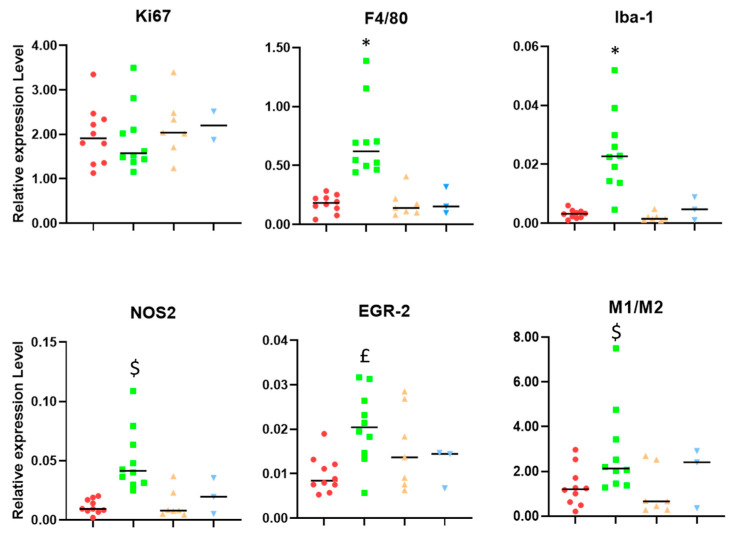
Relative expression levels (related to housekeeping gene expression) of genes encoding for Ki-67, F4/80, Iba-1, NOS2, and EGR-2. The M1 to M2 ratio was calculated with the relative expression levels of characteristic phenotypic markers NOS2 (M1-like) and EGR-2 (M2-like). Groups were as follows, as described in Figure 1: untreated (red), treated-high TRI (green), relapsing (orange), and non-responding (blue). Please see Section 4.3 for an explanation of high/low TRI and its meaning. * = *p* < 0.05 vs. all other groups. $ = *p* < 0.05 vs. control and relapsing;. £ = *p* < 0.05 vs. responding and relapsing. Black lines represent median values.

As can be seen in Figure 2, samples from IMS-TMZ-treated mice mounting successful responses against GB showed an increase in the general GAM-related genes, such as ionized calcium-binding adaptor molecule (Iba-1) and F4/80, while relapsing and unresponsive tumors presented values that were not significantly different from untreated controls. Interestingly, this increase was not linear for M1 and M2-related populations in the case of sustained response, where a switch towards a more antitumoral phenotype was observed (i.e., an increase in NOS2 expression levels and an increase in M1-to-M2 ratios). There was a slight decrease in the Ki-67 expression level, albeit non-significant. 

**Figure 3:**
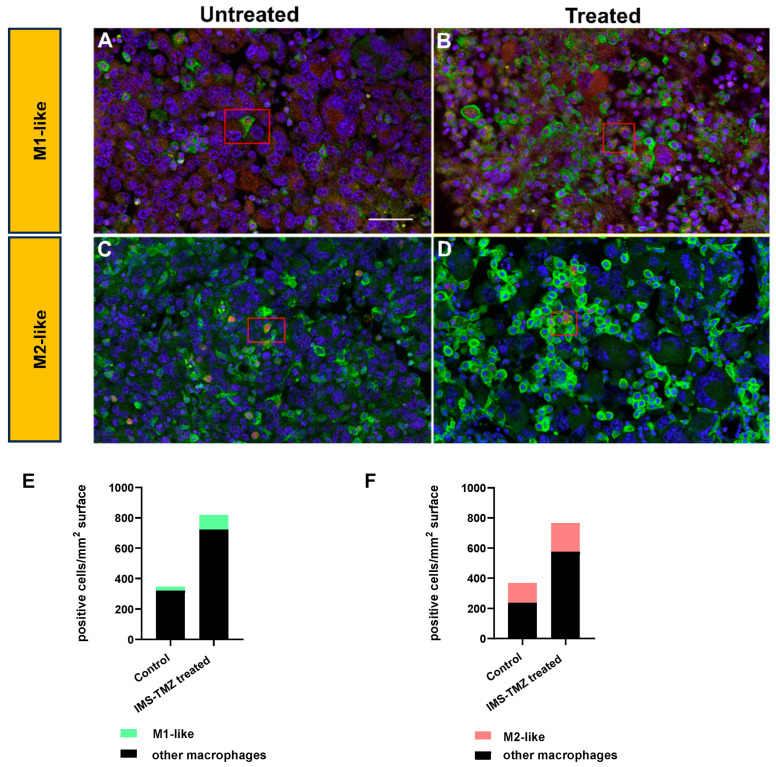
Immunostainings for F4/80, NOS2, and Arg1 to indicate macrophage populations in GB sections from untreated (**A**,**C**) or TMZ-treated and responding (**B**,**D**) mice. Stainings were performed for general macrophage marker F4/80 (green fluorescence, **A**–**D**), for M1 marker NOS2 (red in **A**,**B**), and for M2 marker Arg1 (red in **C**,**D**) for use in double stains. Sections were counterstained for nuclei using DAPI. Double-positive macrophages are indicated in the red boxed areas of (**A**–**D**); the scale bar in (**A**) is 50 μm and is valid for all images. (**E**,**F**) Quantification of macrophage populations based on counting 5 sections in each group. A proportion of M1-like (**E**) and M2-like (**F**) macrophages is provided and significant for M1 (*p* < 0.05) but not for M2-like (*p* = 0.089).

Immunofluorescence studies were performed to analyze whether results from RT-qPCR would reflect macrophage populations. Figure 3 below shows images obtained for untreated and IMS-TMZ-treated high TRI samples for M1 and M2 markers. Such results were aligned to those depicted in Figure 2, namely, we observed both a significant increase for M1-like macrophage populations, whereas the increase in M2-like macrophages was only moderate, so that the M1/M2 ratio overall rose in treated and responding samples compared to the respective untreated controls.

**Figure 4:**
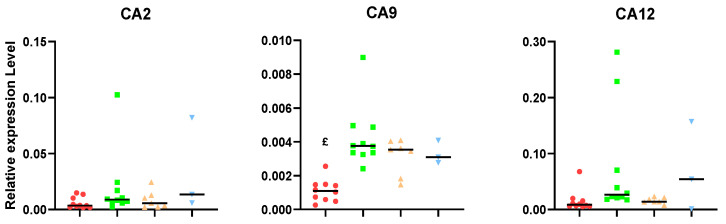
Relative expression levels (relative to housekeeping gene expression) of carbonic anhydrase CA2, CA12, and CA9. £ = *p* < 0.05 vs. responding and relapsing, and a trend to significance (0.05 < *p* < 0.1) vs. unresponsive. Groups were as follows, as described in Figure 1: untreated (red), treated-high TRI (green), relapsing (orange), and non-responding (blue).

Expression levels of carbonic anhydrases (CAs) are dysregulated in GB [21]. Moreover, quantities of lactate generated by tumor cells may vary during response, forcing cells to acquire adaptive mechanisms to avoid excessive acidosis and perturbations in intracellular pH [22,23], and CAs are key elements in this process. Of the 15 CA isoforms expressed in humans, CA2, CA9, and CA12 have been shown to be predominant in GB [24], associated with metabolism, TMZ response, hypoxia (mainly CA9, [25]), cell survival, and proliferation [26]. On the other hand, endothelial CA2 expression seems to be a common phenomenon among high-grade diffusely infiltrating astrocytomas and is also found to correlate with a poor prognosis [27]. Regarding CA2 and 12, no significant changes in expression levels were observed (Figure 4), although there was a trend to an increase in median values of unresponsive tumors, which were 1.5–2.0 times higher than tumors showing sustained response. A different behavior was found in CA9 expression, which was induced in all IMS-TMZ-treated groups, regardless of the response shown, suggesting that hypoxia is present under all these conditions. 

**Figure 5:**
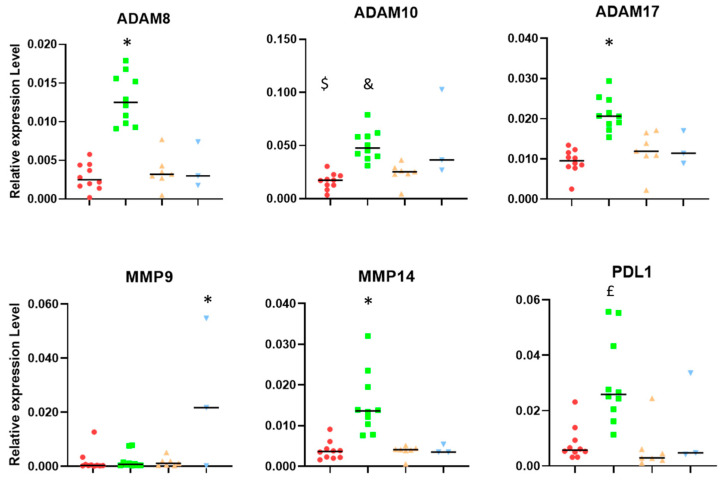
Relative expression levels (related to housekeeping gene expression) of ADAM8, ADAM 10, and ADAM17, and MMP9, MMP14, and PD-L1. Groups were as follows, as described in Figure 1: untreated (red), treated-high TRI (green), relapsing (orange), and non-responding (blue). Please see Section 4.3 for an explanation of high/low TRI and its meaning. * = *p* < 0.05 vs. all other groups. $ = *p* < 0.05 vs. responding and non-responding. & = *p* < 0.05 vs. relapsing. £ = *p* < 0.05 vs. responding and relapsing.

With respect to the metalloproteases analyzed in this study, tumors showing a consistent response to IMS-TMZ have shown increased levels of ADAM8, ADAM10, ADAM17, and MMP14, while other groups did not show significant changes in the expression levels (Figure 5). A different trend was observed for MMP9, which was found to be significantly higher in unresponsive samples. On the other hand, PD-L1 expression was significantly increased in IMS-TMZ-treated samples, either responding or unresponsive, while relapsing samples were not significantly different from control samples. 

**Figure 6:**
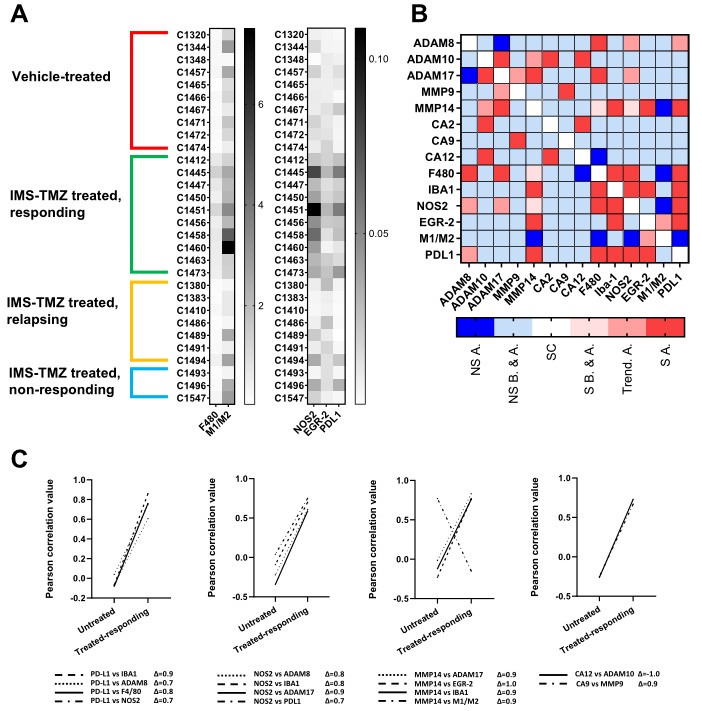
(**A**) Heatmaps for relative expression levels (relative to housekeeping gene expression) of F4/80, Iba-1, NOS2, EGR-2, and PD-L1. The M1 to M2 ratio was calculated with the relative expression levels of the characteristic phenotypic markers NOS2 and EGR-2. Groups were as follows: untreated (red), treated and responding (green), relapsing (orange), and non-responding (blue). Groups were separated into two different heatmaps due to the difference in relative expression levels. (**B**) Heatmap expressing how correlation coefficients change after successful treatment (i.e., treated-responding mice). We assumed that the “control” mouse situation is representative of the pre-treatment situation in all cases. (**C**) Graphs showing the magnitude of change in Pearson correlation coefficient during response to IMS-TMZ treatment for correlations achieving significance, or tending to significance, during response to treatment. Legend for Figure 6B: NS.A. non-significant (loses significance) after therapy; NS B&A, non-significant before and after therapy; SC, self-correlation; S B&A, significant before and after therapy; Trend A. trend to significance after therapy; S.A. achieve significance after therapy.

From Figure 6A, we can visually spot a clustering of high expression levels for microglia/macrophages (F4/80 expression levels), a change in the M1-like/M2-like ratio (NOS2/EGR-2 ratio), and an increase in PDL1 expression in mice treated with IMS-TMZ and responding to therapy. The NOS2 expression also increased in treated and responding samples (Figure 2, Figure 3 and Figure 6A). Thus, it is obvious that there is an overall change in the composition of the tumor mass, which is especially relevant in the case of the GAM population, which could represent up to 40% of the tumor mass [28] and definitely contribute to the registered metabolomic pattern in MRSI acquisition. Moreover, in Figure 6B, we explore the correlations between markers of macrophage populations with molecular signatures that are due to expression levels of MMP9, MMP14, ADAM proteases, and metabolic enzymes of the carbonic anhydrase family and their changes during sustained response to TMZ therapy: some of them achieved significance, while others decreased. One of the correlations that suffered a drastic change in value in samples from mice treated and responding to therapy was the correlation between ADAM8 and PD-L1. Notably, this correlation changed from a negative (−0.07), non-significant value (Figure 6C), to a positive and significant value (0.7), pointing to an aspect worth investigating by hypothesizing that this correlation is functionally relevant in the TME of GB. Consequently, this was further investigated by GL261 cells deficient in the *Adam8* gene.

**Figure 7:**
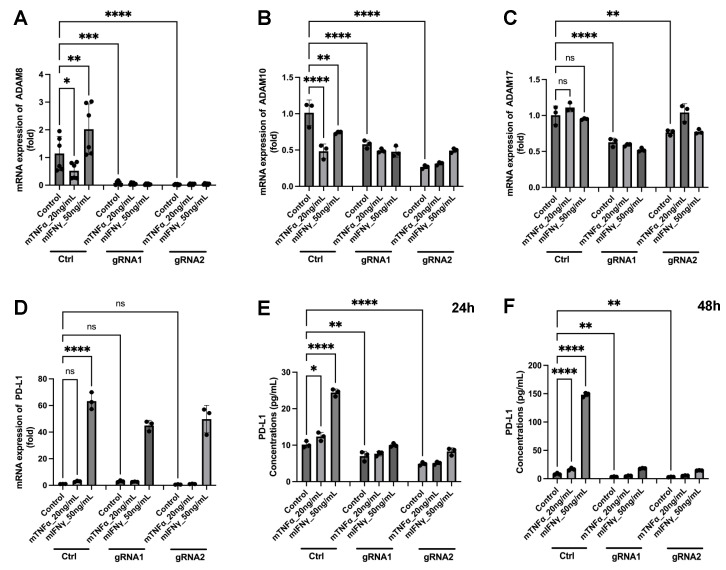
ADAM8 mediates the release of PD-L1 from mouse glioblastoma GL261 cells. (A-D) Expression of ADAM proteases ADAM8, 10, and 17 and PD-L1 in GL261_Ctrl cell, GL261_ADAM8_gRNA1 knockout cell, and GL261_ADAM8_gRNA2 knockout cell. The mRNA expression of ADAM8 (**A**), ADAM10 (**B**), ADAM17 (**C**), and PD-L1 (**D**) after mTNFα (20 ng/mL) and mIFNγ (50 ng/mL) stimulation at 24 h was detected by RT-PCR. The mRNA level of ADAM8 in GL261_Ctrl cells increased after mIFNγ stimulation (**A**). (**E**,**F**) PD-L1, detected by ELISA measurement from the conditioned media of the treated cells from RT-qPCR experiments, was strongly released after mIFNγ stimulation in GL261_Ctrl cells at 24 h (**E**) and 48 h (**F**) time points. Data are presented as mean ± SD, One-way ANOVA was used for analysis. * *p* < 0.05; ** *p* < 0.01; *** *p* < 0.001; **** *p* < 0.0001; ns: not significant.

The observed correlation between expression levels of ADAM8 and PD-L1 but not between those of ADAM10 and ADAM17 and PD-L1 suggests a unique and novel protease-substrate relationship, i.e., by the release of soluble PD-L1 (sPD-L1) from cells (Figure 7). ADAM8 is virtually deleted in GL261 gRNA1 and gRNA2 cells (Figure 7A). In GB, it was reported that IFNγ [29] and TNFα [30] are expressed in the TME. To mimic this situation, we used these cytokines in GL261 cells and, additionally, IFNγ for the polarization of macrophages. Notably, neither mTNFα nor mIFNγ induced activation of ADAM10 (Figure 7B) and ADAM17 (Figure 7C) at the transcriptional level in both WT and KO GL261 cells. In contrast, ADAM8 is significantly upregulated by IFNγ in GL261 cells (Figure 7A). Notably, the knockout of *Adam8* prevented the prominent release of PD-L1 from GL261 cells after stimulation with mIFNγ (Figure 7E,F), which was not attributable to a decreased expression level of the corresponding PD-L1 mRNA (Figure 7D). Proteolytic release of PD-L1 under therapeutic conditions may have implications for therapeutic outcomes. Soluble PD-L1 could potentially interact with the PD-1 receptor on distant T cells in the TME, thereby amplifying the immunosuppressive mechanism (see also Section 3.4).

**Figure 8:**
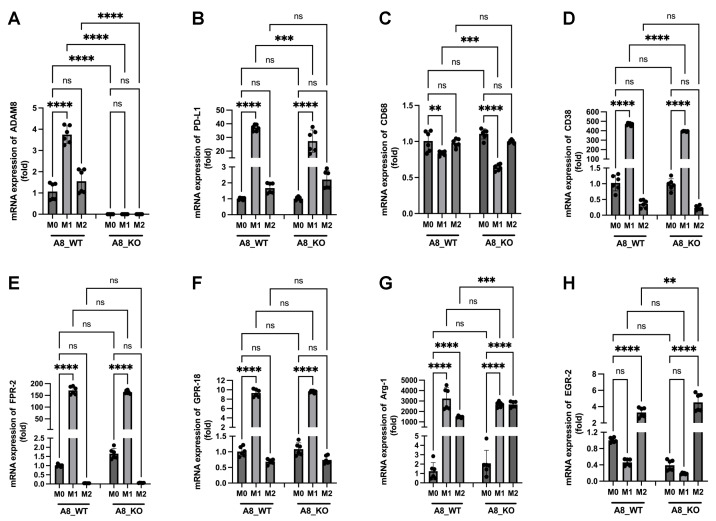
Expression of M1 and M2 polarization macrophage markers in macrophages isolated from the bone marrow of A8 WT mice and A8 KO mice. (**A**) ADAM8, (**B**) PD-L1, (**C**) CD68, (**D**–**F**) M1-polarization macrophage marker, CD38, FPR2, and CPR-18, (**G**,**H**) M2-polarization macrophage marker, Arg-1 and EGR-2 after LPS, mIFNγ, and mIL4 stimulation at 24 h were detected by RT-PCR. Data are presented as mean ± SD. A one-way ANOVA was used. ** *p* < 0.01; *** *p* < 0.001; **** *p* < 0.0001; ns: not significant.

**Figure 9:**
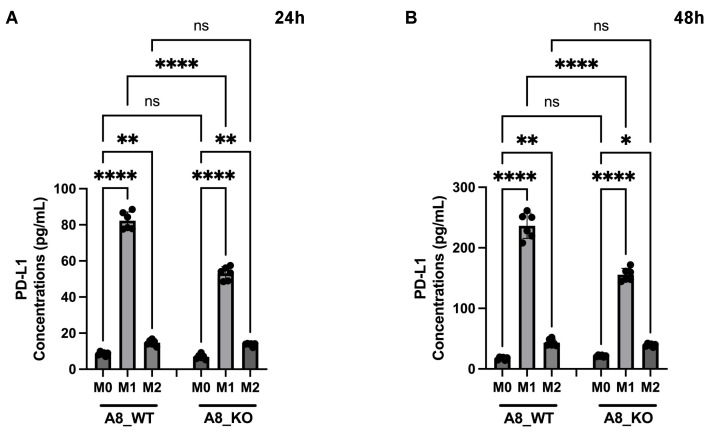
PD-L1 release is dependent on M1 polarization in ADAM8-expressing macrophages. PD-L1 release after LPS, mIFNγ, and mIL4 stimulation at 24 h (**A**) and 48 h (**B**) was detected by ELISA measurement. Data are presented as mean ± SD. A one-way ANOVA was used for analysis. * *p* < 0.05; ** *p* < 0.01; **** *p* < 0.0001; ns: not significant.

Data in Figure 6B,C show that PD-L1 expression achieved significance in correlation with microglia/macrophage markers, as well as M1-like and M2-like specific markers. Such an expression pattern was described by others [31], including GAMs [32], but the role of ADAM8 in this scenario was, to our knowledge, not previously studied. To analyze this correlation on the cellular level, we isolated bone-marrow-derived macrophages from WT C57/BL6 and ADAM8-deficient mice and proceeded to M1 or M2 polarization, followed by the analyses of general macrophage marker, specific M1 and M2 markers, as well as ADAM8 and PD-L1 release. In Figure 8, we can appreciate that polarization was successful and that a knockout of ADAM8 significantly reduced PD-L1 release by macrophages, mostly performed by the M1-like subtype. However, the ADAM8 effect on PD-L1 shedding was much more pronounced in GB cells than in macrophages, suggesting that other proteases in addition to ADAM8 might contribute to PD-L1 shedding in these cells, one of which could be MMP14. To address the main question of whether PD-L1 release is dependent on ADAM8 expression, we utilized LPS + mIFNγ as well-established inducers of M1-like macrophage polarization and mIL-4 as inducers of M2-like macrophage polarization [33]. First, we tested the ADAM8 expression in WT and *Adam8* KO macrophages. ADAM8 is completely absent in the macrophages of KO mice compared to WT mice (Figure 8A). Next, we focused on different macrophage markers, including general macrophage markers, CD68; M1 polarization markers, CD38, FPR-2, and GPR-18; and M2 polarization markers, Arg1 and EGR-2. CD68, as a general macrophage marker, has a high expression in macrophages; however, no significant differences were observed in M1-like and M2-like macrophages and in WT and KO mice (Figure 8C). In many M1 candidates, we focused on CD38, FPR-2, and GPR-18 due to their predicted membrane expression patterns that could lead to improved M1 detection and sorting by flow cytometry [34]. CD38 was most significantly upregulated in M1 compared to M0 or M2 (Figure 8D). FPR-2 and GPR-18 were also up-regulated by 10-150-fold (Figure 8E,F). Two independent M2-like markers, EGR-2 and Arg1, were strongly induced in M2, with over a four-fold increase (Figure 8H), and Arg1 is highly expressed not only in M2 macrophages but also in M1 macrophages, at least at the transcriptional level (Figure 8G). Next, we also analyzed the PD-L1 changes at different conditions in ADAM8 WT and ADAM8 KO macrophages. PD-L1 was most significantly upregulated in M1 compared to M0 and M2 at both the transcriptional levels (Figure 8B) and protein release (Figure 9A,B). Although PD-L1 was also released in M1 polarized macrophages in KO mice, PD-L release was much more pronounced in WT mice (Figure 9A,B), showing that deficiency in the *ADAM8* gene partly prevented the release of PD-L1, and other ADAM proteases could be involved in PD-L1 shedding from macrophages.

Since the preclinical GB samples described in Figure 2, Figure 3, Figure 4, Figure 5 and Figure 6 were obtained guided by the metabolomics (MRSI)-based biomarker reflected in nosological images, we assessed such data to investigate the metabolic pathways affected and provide a possible explanation for such changes. It is worth mentioning that inclusion criteria for those samples also relied on the volumetric behavior of the tumor, i.e., control samples presented fast growth and IMS-TMZ-treated, responding samples presented either growth arrest or tumor volume decrease. Both non-invasive criteria were used for sample collection (described in Section 4.3) and further analyses in order to ensure robust sampling. Multi-slice MRSI spectra originated from control and IMS-TMZ-treated, responding mice (dataset previously published in [16]), and the expected nosological images were obtained, namely: control (untreated) mice presented mostly spectral features comparable to proliferative GL261 GB, while responding mice pixels were mostly classified as GL261 GB responding to therapy. The paradigmatic spectra of responding vs. control (or unresponsive) conditions are shown in Supplementary Figure S3, as well as the highlighted differences between both situations. Please note that those are not individual spectra or average spectra, but a mathematical extraction of the patterns (named sources) corresponding to several inputs for each situation [20]. Representative spectra of zones from mice illustrated in Figure 1B can be found in Supplementary Figure S4. 

**Figure 10:**
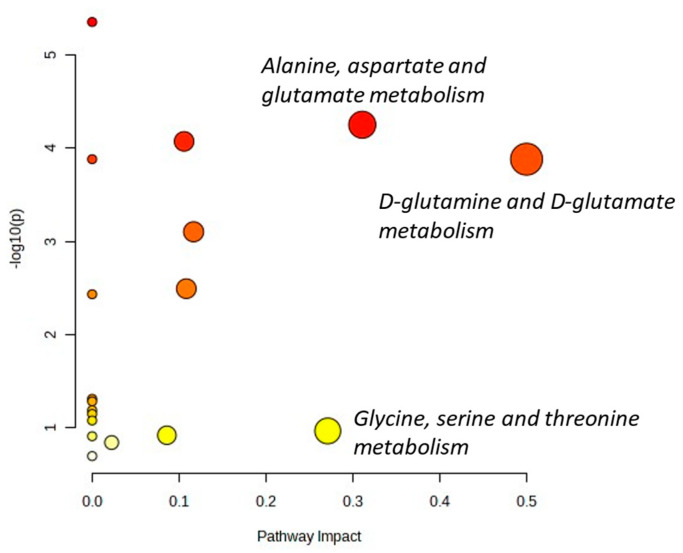
Metabolome view showing all matched pathways according to the *p* values from the pathway enrichment analysis and the pathway impact values from the pathway topology analysis. The main potentially affected pathways are shown.

As depicted in the aforementioned figures, the main differences arise from lactate, alanine, glutamate, glutamine, glycine, and polyunsaturated fatty acids (PUFA). Metaboanalyst 5.0 was used to assess the main metabolic pathways in which such metabolites were involved. With the exception of PUFAs, which are usually related to apoptosis taking place during treatment [35], the combination of other metabolites suggests deregulation of some important metabolic pathways involved in cancer cell processes, such as glycine/serine metabolism [36] or glutamine since cancer cells modify the consumption and processing of glutamine to sustain cell growth and proliferation [37] (Figure 10). Last but not least, the change in GAM phenotype might also change the overall metabolic landscape of the tumor microenvironment [38], and metabolites such as alanine could be related to such events [39]. Overall, the data suggest that in vivo metabolomics can be of potential value in the noninvasive assessment of TME-related changes. 

## 3. Discussion

The influence of the TME and the associated changes during response to therapy are acknowledged in the current literature, but the translation/use of such findings in therapy is not yet optimal [40]. Accordingly, in this work, we relied on a GB preclinical model, with its tumor progression guided by non-invasive approaches, such as MRI and MRSI, to obtain samples, allowing detailed studies of the tumor microenvironment changes related to therapy. In addition, we also wanted to correlate such changes with the metabolomics pattern changes observed in MRSI acquisitions, with the intention of providing a better understanding/explanation of the surrogated therapy response biomarker, which proved to be linked to therapy response to different approaches in this GB model [8,15,20]. Still, we extended our investigation previously started in [16], which was restricted to control and IMS-TMZ-treated, responding samples, to encompass samples from mice relapsing after transient response and samples from non-responsive mice, in addition to expanding the gene expression levels analyzed, as well as including other experimental approaches to determine the microenvironmental changes. 

### 3.1. Microglia/Macrophage Populations Present Changes in Their Infiltration Behavior and Phenotypic Characteristics during Successful Response to IMS-TMZ Treatment

Studies with RT-PCR in samples obtained from the four mouse groups were analyzed for the expression levels of microglia/macrophage and tumor cell-related genes (F4-80, Iba-1, Ki-67, EGR-2, NOS2, and PDL1), metalloprotease-related genes (ADAM 8, 10, and 17, and MMP9 and 14), and metabolic-related genes, such as carbonic anhydrases (CA2, 9, and 12), to represent hypoxia-inducible genes. 

Figure 2 shows a trend to lower average Ki-67 values in IMS-TMZ-treated responding samples when compared to control, relapsing, and unresponsive groups, although this trend is non-significant. Ki-67 is a protein that is widely used as a marker for cell proliferation, although other authors state that it is more involved in organizing heterochromatin and that cells lacking Ki-67 can indeed proliferate [41]. Moreover, Ki-67 has been reported to have different functions related to cell transformation, tumor growth, metastasis, drug sensitivity, and maintenance of stem cell features [42]. The same authors also point out that, on the other hand, its expression makes the tumor more visible to the immune system. Still, we must take into account that the microglia/macrophage population, which is significantly high in GB tumors, may also proliferate and contribute to the overall Ki-67 expression level [43]. Since we cannot determine the origin of the Ki-67 expression in our samples (either tumor cells or GAMs) through RT-PCR analysis, the lowering trend observed in tumor cells upon response to therapy [8,20] could be counteracted by an increasing trend from the macrophage population. On the other hand, the global Ki-67 expression level was essentially comparable in control, relapsing, and unresponsive tumors.

F4/80 and Iba-1 (ionized calcium-binding adaptor molecule 1) are well-recognized markers for microglia and macrophage identification and showed significant increases in samples from IMS-TMZ-treated responding mice in comparison with the other groups, with an 8- and 4-fold increase in their expression levels (Figure 2), respectively. Some chemotherapeutics have been described to have immunomodulatory effects through immunogenic cell death, and such effects can lead to an increase in certain macrophage populations [44], and specifically, TMZ was described, both by us and other authors, to have an impact on the TME [45,46,47]. These results also agree with our findings described in immunostained samples, showing an increase in the F4/80-positive population in TMZ-treated responding mice [9]. The increase in macrophage population itself is expected, but the outcome of tumor progression may vary according to the prevailing phenotype. Here, we have observed a significant (3.5-fold) increase for the M1-like gene marker (NOS2) in IMS-TMZ-treated responding samples, accompanied by a more discrete, non-significant 1.7-fold increase in the M2 marker gene (EGR-2). Altogether, this indicates that there could be a switch in the M1-to-M2 ratio in responding samples, albeit non-significant, pointing towards a stronger macrophage-based antitumoral action, which might have an impact on tumor growth arrest or volume decrease [11,48]. Moreover, changes in phenotype prevalence from M1 to M2 were reported to be associated with tumor progression in solid tumors [49,50]. Specifically regarding GB, it has been described that predominant M1 polarization was associated with a better overall prognosis [11] and that the M1/M2 ratio was correlated with the survival rate after TMZ treatment [51]. Moreover, our results suggest that lower M1/M2 ratios (i.e., pointing to a prevalence of M2 phenotype when comparing to IMS-TMZ-treated responding tumors) are associated with a “lack of response”, either starting from the beginning (non-responding) or after transient response (relapsing), or associated with active proliferation in control mice. In all instances, there were no significant differences between them, and values were mostly aligned between control (untreated), relapsing, and unresponsive tumors. The difference between control (untreated) and TMZ-treated (responding) tumors, however, is obvious in the staining patterns (Figure 3) and confirms the qPCR results (Figure 2). Overall, the population of M2-like macrophages is higher than that of M1-like macrophages. Using the M1 marker NOS2, staining was weak and slightly increased in treated tumors, so immunostaining might reflect the increase in the M1/M2 ratio under treatment as seen for qPCR markers.

### 3.2. Carbonic Anhydrase Results Suggest That Unresponsive Tumors Could Overexpress This Gene

CA2 is a highly active cytosolic isoform and has the fastest catalytic rate of the α-CAs enzymes that catalyze the reversible interconversion of carbon dioxide (CO_2_), bicarbonate (HCO_3_^−^), and protons (H^+^), which are involved in various physiological processes, such as pH homeostasis, respiration, etc. [52]. Moreover, we have previously shown that CA2 is a downstream target gene of Bcl-3 for mediating TMZ resistance [13]. Furthermore, the most relevant carbonic anhydrases for GB, CA9 and CA12, were described as tumor cell-intrinsic carbonic anhydrases in GB and other tumor entities [24,53]. Importantly, the activation of CA9 expression depends on the HIF-1α transcription factor activation cascade [54,55], which has been identified as a hypoxia-dependent gene that is relevant for the stem cell niche in GB, in which GB cells or GSCs can survive tumor-targeted therapies [55,56]. Also, CA12 and P-gp, an efflux pump that recognizes a broad spectrum of chemotherapeutics as substrates, including TMZ, are co-expressed in GSCs derived from GB patients with a low response to TMZ, which supports the idea that CA12 may represent another metabolic factor in GB patients resistant to TMZ treatment [57]. Accordingly, we have studied the expression of this set of genes in our murine samples and found that a trend toward higher values is found in unresponsive samples, which is in agreement with an intrinsic resistance to treatment in such samples. 

### 3.3. Changes in the Expression of Metalloproteases Can Shape TME, and Relevant Differences Are Found during Response to IMS-TMZ

In addition to the significant role described for GAM polarization through the M1 or M2 phenotype and the PD-1/PD-L1 axis in the response to therapy, it has been described that zinc-dependent proteases of the metzincin superfamily, such as ADAMs and MMPs, are important modulators of the TME, affecting aspects of tumorigenesis and signaling pathways by cleaving cytokines, chemokines, and growth factors [58,59,60,61]. These protease families have been described as being related to several biological processes, such as inflammatory responses, immune regulation, angiogenesis, cell migration and proliferation, apoptosis, and tissue repair, among others. 

Thus, since ADAMs and MMP proteases may act as important modulators of the TME, the distinct populations of GAMs might also be correlated with specific protease profiles in GB [62,63]. Investigation on human GB patients demonstrated expression of proteases ADAM8, 10, and 17, and MMP9 and 14, which are associated with the occurrence of GAMs and support their functions, e.g., shedding of EGF-R by ADAM17 can direct cancer cell invasion [64], ADAM10 can regulate macrophage survival [65], whereas ADAM8 in macrophages increases MMP9 levels in co-cultured tumor cells [66] so that these ADAM proteases are associated with GAM functions and directly affect the nature of the TME [67,68]. Within the TME, all ADAM proteases investigated here (8,10,17) are expressed not only in cancer but also in tumor-infiltrating immune cells, again suggesting that they are part of tumor–immune cell communication.

Accordingly, we wanted to analyze samples from IMS-TMZ-treated mice either responding or non-responding to treatment, as well as relapsing and vehicle-treated tumors, using RT-PCR in order to characterize the different subpopulations of GAMs and PD-L1 gene expression and the molecular profile of different protease genes known to be associated with GB progression (ADAM8, ADAM10, ADAM17, MMP9, and MMP14). Furthermore, we studied the correlation between GAM population, both M1/M2 subtypes, and metalloprotease expression. The metalloprotease profiles were also associated with the expression of PD-L1 in order to provide information on their role in immunosuppression in the TME. Figure 5 summarizes the results obtained for metalloproteases and PD-L1 expression. Consistent response to IMS-TMZ treatment was accompanied by an increase in the expression level of ADAM8, ADAM 17, and MMP14, which was significant in comparison with other groups. This trend was less clear in the case of ADAM10, while MMP9 was found to have significantly increased in the unresponsive group. The expression level of PDL1 was also notably higher in samples responding to IMS-TMZ therapy, albeit non-significant. PD-L1 expression level is considered a major prognostic biomarker for immune therapy in many cancers, such as renal carcinoma, colorectal cancer, lymphoma, head and neck carcinoma, bladder cancer, hepatocellular carcinoma, and metastatic colorectal cancer, but its predictive value in gliomas is still controversial [69], although some meta-analysis studies point to a prognostic value [70]. 

ADAMs 8, 10, and 17 have been described as being involved in maintaining the malignant phenotype of glioma cells and being linked to their migration, invasion, or proliferation. Moreover, other authors stress that these metalloproteases could also modulate the immunophenotype of glioma-initiating cells, and their presence would contribute to generating an immunosuppressive phenotype [71].

### 3.4. Correlation between the Expression Levels of Different Genes Showed Unexpected Links

We have explored, through Pearson correlation tests, the existing links between different gene expression levels and, in addition, possible changes in correlation triggered by IMS-TMZ treatment with sustained response to therapy (Figure 6). These results suggest that there are groups of genes with related changes triggered by therapy and followed by a sustained response. As such, Figure 6B shows a combined representation of how the correlation between the studied gene expression levels changed during response to IMS-TMZ therapy, i.e., whether specific correlations achieved significance, lost significance, or remained unchanged. Some of the correlations were expected, such as PDL1 vs. macrophage-related genes, since PD-L1 is known to be expressed in a variety of cell types, including macrophages [72], also reported in brain macrophages [73]. 

Proteolytic processing of PD-L1 was described to be associated with metalloproteases ADAM10 and ADAM17 [74], so a correlation of their expression levels was expected in IMS-TMZ-treated samples. Surprisingly, our results revealed a different scenario, and the PDL-1 expression level increase was found to be rather correlated with ADAM8 levels (Figure 6B,C), suggesting the involvement of ADAM8 in PD-L1 processing in GB. To our knowledge, PD-L1 processing had not been associated with ADAM8 before; thus, we questioned whether a deeper investigation in this manner could unravel the role of ADAM8 in this context. A different trend in ADAM8 expression in GB samples from patients had already been hinted at by us [11]. This potential relationship between ADAM8 and PD-L1 expression was demonstrated by the knockout of *Adam8* in GL261 cells (Figure 7). Notably, a deficiency of ADAM8 prevented the release of PD-L1. In this regard, the sPD-L1 status would have an impact on its ability to diffuse away from the original PD-L1-expressing cells, extending its effects to the entire TME and beyond, or perhaps diluting them below a relevant concentration for the target effect. Thus, sPD-L1 could have an effect on the ability of approaching lymphocytes to infiltrate the GB tumor in an active, energized form only when its TME concentration is high enough. On the other hand, the decrease in membrane-bound PD-L1 would diminish the ability of tumor cells in ADAM8-high regions to compromise local lymphocyte attack. Those findings hint at a new potential therapeutic target that could facilitate the “warming” of cold GB tumors.

### 3.5. Microenvironment Changes during Sustained Response to IMS-TMZ Were Translated into Metabolomic Changes Noninvasively Assessed with MRSI

The tumor progression in response to IMS-TMZ therapy was assessed in all studied groups through volume calculation using T_2_-weighed MRI. However, there is a special interest in both (i) assessing tumor microenvironment features associated with successful responses to therapy and (ii) investigating non-invasive approaches that could provide early hints of successful microenvironment changes triggering responses, since it is well known that MRI alone lacks precision in early response stages due to phenomena such as pseudo-progression and pseudo-response. As such, metabolomic approaches, such as magnetic resonance spectroscopy, could be of great help. Moreover, MRSI (spectroscopic imaging) combines metabolomic and spatial information, and the use of machine learning approaches helps to unravel the rich information contained in magnetic resonance spectra and the subtle changes derived from reprogramming both tumor cells and GAM different phenotypes. In this work, we used our previously trained classification system for detecting responses in GL261 GB for determining the time point for tumor-bearing mouse euthanasia in order to obtain samples for further validation. Strict inclusion criteria ensured that suitable samples were obtained and displayed a consistent response. Accordingly, since mice were euthanized for sample obtention, it was not possible to perform a longitudinal assessment and have complete information about the outcome. However, our initial premise relies on similar changes in sustained or transient response to therapy, which, in the case of relapsing samples, are either reverted, suppressed, or counteracted by tumor growth and increased intracranial pressure. 

Our results show that samples obtained from GL261 GB-bearing mice treated with IMS-TMZ and showing consistent response to therapy presented a collection of changes consisting of an increased GAM population, a switch to a prevailing antitumoral GAM phenotype, an increase in metalloproteases such as ADAM8/10/17 and MMP14, and an increase in PD-L1 expression. The increase in expression of ADAM8 was remarkable, being five-fold higher in IMS-TMZ-treated responding samples in comparison with controls. This was also accompanied by a higher expression of CA9, although this change in expression was not a distinctive trait in comparison with other treated groups. Overall, and since the tumor mass has a relevant contribution both from tumor cells and GAMs, the metabolic reprogramming in such cells is certainly being reflected in MRSI-acquired data and, if proven consistent, could be used for early assessment of response (or lack thereof). This comprehensive view with correlations between spectroscopic and molecular data reflecting the cellular composition of GB is a novel approach and deserves further investigation to validate these findings.

### 3.6. A New Therapeutic Target Emerges from the ADAM8/PD-L1 Relationship

The correlation between ADAM8 and PD-L1 expression levels was investigated for several tumor types using the TIMER 2.0 resource (http://timer.cistrome.org/, accessed on 30 October 2023) and showed a positive value for 33 out of the 40 tumor types investigated, including GB (Supplementary Figure S5). However, it is worth noting that in our case, samples from untreated GL261 GB showed a negative correlation between these two gene expressions, which turned strongly positive during sustained response to IMS-TMZ therapy. In previous studies, we have described the role of ADAM8 as a protease involved in TMZ-induced chemoresistance and enhanced invasion of GB cells, already hinting at a possible therapeutic target [75], but in light of the results gathered in the present work, a combination of ADAM8 targeting and anti-PD-1 immunotherapy may be even more effective and is one possible mechanism by which the tumor microenvironment in GB could be made more susceptible to immunotherapy. 

## 4. Materials and Methods

### 4.1. GL261 Cell Culture

GL261 mouse glioma cells were obtained from the Tumor Bank Repository at the National Cancer Institute (Frederick, MD, USA) and were grown as previously described [76]. Briefly, cells were grown in RPMI-1640 culture medium, which was supplemented with 2.0 g/L NaHCO_3_, 0.285 g/L L-glutamine, 10% fetal bovine serum, and 1% penicillin/streptomycin solution.

### 4.2. Preclinical GL261 GB Model and IMS-TMZ Treatment 

Mice were obtained from Charles River Laboratories (France) and housed at the animal facility of the *Universitat Autònoma de Barcelona* (*Servei d’Estabulari*), https://sct.uab.cat/estabulari/content/presentaci%C3%B3.html (accessed on 30 October 2023). Animal welfare was assessed weekly following a supervision protocol for brain-tumor-bearing animals in order to evaluate the severity of the symptoms generated by the growing masses. All studies were approved by the local ethics committee (CEEAH 3665), following European, national, and autonomic regulations. A total of 41 C57BL/6 female mice (weighing 21.4 ± 1.4 g, aged 15.5 ± 2.8 weeks) were used for this work. Prior to tumor generation, mice were maintained for 3 weeks in an enriched environment [77], and during this time, no procedure was carried out. Afterwards, the GL261 glioma generation was carried out through intracranial stereotactic injection of 1 × 10^5^ cells into the caudate nucleus, as described in [76]. For animal treatment, TMZ (Sigma-Aldrich, Madrid, Spain) was dissolved in 10% dimethyl sulfoxide (DMSO) in a saline solution (0.9% NaCl). Mice were divided into treated and control groups, and IMS-TMZ treatment (i.e., 60 mg/Kg every 6 days) was administered intragastrically using an oral gavage, starting at day 11 post-injection (p.i.), while control mice received 10% DMSO vehicle. [76].

### 4.3. MR Data Acquisition (MRI and MRSI), Processing, and Postprocessing, and Murine Tumor Sample Collection

All MRI/MRSI studies were carried out at the joint nuclear magnetic resonance facility of *Universitat Autònoma de Barcelona* and *Centro de Investigación Biomédica en Red-Bioingeniería, Biomateriales y Nanomedicina* (CIBER-BBN) (Cerdanyola del Vallès, Spain), Unit 25 of NANBIOSIS (www.nanbiosis.es, accessed on 30 October 2023). Data were acquired with a 7T Bruker BioSpec 70/30 USR spectrometer (Bruker Biospin GmbH, Ettlingen, Germany). The MRI and MRSI parameters were essentially as described in [9]. A brief description of the MR-based methods will be given below, while a detailed description of equipment, acquisition, and processing parameters can be found in the Supplementary Information File. 

MRI acquisition: High-resolution coronal T_2_w images were acquired using a Rapid Acquisition with Relaxation Enhancement (RARE) sequence in order to confirm suitable tumor presence/volume and to monitor its evolution stage, with repetition time (TR)/effective echo time (TEeff) = 4200/36 ms. 

MRSI acquisition: MRSI was acquired with a point-resolved spectroscopy (PRESS) localization sequence at 14 ms echo time (TE), and grids were positioned individually across the tumor, using a reference T_2_w high-resolution MRI, as described in [8]. In general, three to four MRSI grids were enough to cover the whole tumor. The matrix size was 10 × 10 or 12 × 12. Shimming was performed individually for each MRSI grid, which was carefully placed, ensuring that the volume of interest (VOI) included most of the tumor area and also normal/peritumoral brain parenchyma.

MRI/MRSI processing/postprocessing and nosological imaging generation: Tumor volume calculations were undertaken through manual segmentation in T_2_w high-resolution horizontal images. MRSI data were post-processed essentially as described in [8]. Data were pre-processed at the MR workstation with ParaVision 5.1 and then post-processed with 3D Interactive Chemical Shift Imaging (3DiCSI) software package version 1.9.17 (courtesy of Truman Brown, PhD, Columbia University, New York, NY, USA). Data were exported to ASCII format, and each spectrum of the MRSI matrix was aligned with a home-developed processing module, Dynamic MRSI (DMPM), running over MatLab 2013a (The MathWorks Inc., Natick, MA, USA). The 0 to 4.5 ppm region of each spectrum was normalized to unit length and exported in ASCII format. A previously developed semi-supervised machine learning (ML) methodology based on non-negative matrix factorization (Convex-NMF) [20] was used for classifying pixels of each matrix into normal brain parenchyma, actively proliferating tumors, and tumors responding to treatment, and for calculating nosological maps representing the spatial response to treatment based on the metabolomics spectral pattern. Green is used when the GB responding to treatment source contributes the most; blue is used for normal brain parenchyma; red is used for actively proliferating GB; and black is used for an undetermined tissue class.

Tumor responding index (TRI) calculations: According to the approach described by us in [8], and in order to measure the extent of response to treatment using the nosological images, the following parameter, TRI, was calculated:

(1) $$\mathrm{TRI}=\frac{\mathrm{Tumor}\;\mathrm{responding}\;\mathrm{pixels}}{\mathrm{Total}\;\mathrm{tumor}\;\mathrm{pixels}}\times 100$$

TRI is defined as the percentage of tumor pixels (over total tumor pixels) classified as responding by our previously described ML approach (green pixels in the nosological image). We established thresholds for TRI in order to guide sample obtention for the treated-responding and untreated/control mice, as described in [16], namely, higher than 65% for treated/responding cases and as close to 0% as possible for control cases (see also Figure 1B). This information was combined with an adapted set of RECIST criteria [19] used to classify cases into progressive disease (PD), partial response (PRe), or stable disease (SDi). See Supplementary Information for more details on the adapted RECIST criteria and an example of TRI calculation. 

Sample obtention: In this work, 4 different groups were studied, 3 of which were treated with IMS-TMZ schedules. For the untreated/control and treated/responding groups, sample obtention was guided by TRI values coming from MRSI and ML studies, combined with volumetric measurements coming from MRI data. Inclusion criteria were described in detail in [16], and regarding TRI, extreme values were searched for, as stated in the previous subsection. For relapsing and unresponsive groups, sample obtention was guided by volumetric information obtained from MRI data.

Samples for RT-PCR: At the defined time points, mice were euthanized by cervical dislocation, and tumors were dissected and stored in liquid N_2_ until further processing (Section 4.4). 

Samples for immunofluorescence: (a) Perfusion: At the experimental endpoint, animals were deeply anesthetized with 100 mg/kg i.p. of ketamine (Imalgene; *Merial laboratorios*; Barcelona, Spain) and 0.5 mg/kg medetomidine (Domtor; Orion Corporation; Espoo, Finland), and perfused intra-cardially with tyrode solution (NaCl, KCl, CaCl_2_, MgCl_2_, NaH_2_PO_4_, NaHCO_3_, D-Glucose, and H_2_O) and with 4% paraformaldehyde in 0.1 M PBS using a peristaltic pump. (b) Extraction of brain tissue: Once the perfusion was completed, brains were immediately removed and post-fixed for 24 h at 4 °C in 4% paraformaldehyde. After this, they were placed in 0.1 M PBS with sodium azide under the same storage conditions. Subsequently, brains were cut into 60 μm thick serial sections in a vibratome (Leica VT1000S, Leica Inc., Wetzlar, Germany) in the coronal plane to obtain a representative sample of all levels of the tumor. The 60 μm slices were collected starting from the anterior-most portion of the tumor and sequentially placed with a soft-bristled brush inside a sterile 24-well box (Nalgene, Rochester, NY, USA), previously filled with 0.1 M PBS and 0.1% sodium azide. Sections were placed one by one in six series; this allowed 9–10 representative sections of tumor to be obtained for each well. The tissue was stored in 0.1 M PBS with 0.1% sodium azide until use.

It is worth clarifying that n = 10 responding IMS-TMZ-treated mice and n = 10 IMS-vehicle-treated (control) mice in the RT-PCR group are the same individuals described in [16] regarding M1/M2/PD-L1 profiling. In this work, we have worked towards enlarging the cohort of mice, including individuals belonging to relapsing (after transient response, n = 7) and unresponsive groups (n = 3), and we have also enlarged the IMS-TMZ-treated responding group with n = 2 additional cases. 

### 4.4. RNA Isolation, cDNA Synthesis, and RT-PCR from Mouse Brain Tissue

Analysis of relative mRNA expression levels of F4/80, NOS2, EGR-2, CD274 (PD-L1), ADAM8, ADAM10, ADAM17, MMP9, and MMP14 was carried out in all collected samples. RNA isolation and cDNA synthesis were performed following previous descriptions [16]. For F4/80, NOS2, EGR-2, and PD-L1 RT-PCR analyses, the experimental steps were as in [16]. For ADAM8, ADAM10, ADAM17, MMP9, and MMP14 RT-PCR analyses, 2 ng of cDNA were used, all reactions were performed twice, and results were averaged. The RT-PCR was performed using a StepOne-Plus Real-Time PCR instrument (Applied Biosystems, Waltham, MA, USA; Thermo Fisher Scientific, Dreieich, Germany) and SYBR Green in the form of the Precision FASTMasterMix with ROX (Primer Design, Southampton, UK). PCR amplification reactions were carried out in 20 μL reaction volumes, and the protocol consisted of an initial denaturation at 95 °C for 10 min, followed by 40 amplification cycles at 95 °C for 15 s and 60 °C for 1 min. The mRNA for the acidic ribosomal protein RPLP0 (XS13) served as an internal reference gene for all RT-PCR reactions. Cycle time (Ct) was calculated by StepOne Software v2.0 (Applied Biosystems). For each gene, the 2^−ΔΔCt^ method [78] was performed to analyze relative quantities. 

### 4.5. Construction of Stable GL261 ADAM8 Knockout Cells

GL261 cells were transfected with two different gRNAs using the CRISPR/Cas9 Knockout/Knockin Kit from OriGene (Cat No. # KN500845). All steps used for the transfection of cells were performed as described previously [66,79]. Cloned cells were selected through treatment with puromycin (1 μg/μL, 58-85-2, InvivoGen, San Diego, CA, USA). The cell status was confirmed by RT-PCR. GL261 WT cells were used as a control.

### 4.6. RNA Extraction, cDNA Transcription, and RT-PCR from GL261 Cells

A total of 5 × 10^5^ GL261_WT, GL261 ADAM8 gRNA1 Knockout, and GL261 ADAM8 gRNA2 Knockout cells were seeded in 6-well plates overnight, changed with fresh medium containing mTNFα (50 ng/mL, 315-01A, PeproTech, Hamburg, Germany) and mIFNγ (20 ng/mL, 315-05, PeproTech), and incubated for 24 h and 48 h. Cells were washed 3 times with PBS. Conditioned medium was used for ELISA measurement as described in Section 4.7.

Total RNA was isolated by QIAzol reagent (79306, Qiagen, Hilden, Germany). The detailed steps of RNA extraction were as described before [66]. Two μg of RNA was reverse transcribed into cDNA with the RNA to cDNA EcoDry™ Premix kit (Takara Bio Inc., Kusatsu, Japan) according to the manufacturer’s instructions. The RT-PCR was performed using iTaqTM Universal SYBR^®^ Green SuperMix (Bio-Rad, Feldkirchen, Germany); the target primers were acquired in Qiagen; and XS_13 was used as a housekeeping gene (forward: 5-TGGGCAAGAACACCATGATG-3; reverse: 5-AGTTTCTCCAGAGCTGGGTTGT-3). The fold changes of gene expression relative to the control group were calculated with 2^−ΔΔCт^ [78].

### 4.7. ELISA Measurements 

The conditioned medium was collected as described in Section 4.6 and Section 4.8; the supernatant was stored at −20 °C after centrifugation and subjected to PD-L1 ELISA detection according to the manufacturer’s protocol (DY1019-05, R&D Systems, Minneapolis, MN, USA). Firstly, a 96-well microplate was coated with the diluted capture antibody, maintained overnight, and washed three times with washing buffer. The plate was blocked with reagent buffer (1% BSA) for 1 h. Afterwards, standards and samples were incubated for 2 h at room temperature, and detection antibodies were added with an incubation time of 2 h. Finally, incubation with Streptavidin-HRP (horseradish peroxidase) was performed for 20 min, plus 20 min of incubation with substrate solution at room temperature, avoiding direct light exposure. After adding Stop Solution, the plate was measured by the ELISA reader set to a wavelength of 450 nm.

### 4.8. In Vitro Polarization of Macrophages 

Macrophages were isolated separately from the bone marrow of A8 (*Adam8*) WT mice and A8 KO mice. Cells were cultured in Petri dishes in a complete medium with M-CSF (20 ng/mL, #315-02, PeproTech, Hamburg, Germany). Afterwards, 5 mL of the complete medium with M-CSF was added on day 3, and after 7 days of culturing the macrophages, cells were seeded on 6-well plates. Cells received media alone for classical M0 macrophages, or were activated to M1-polarized macrophages with lipopolysaccharide (LPS, 100 ng/mL, Sigma-Aldrich, Munich, Germany) + interferon gamma (mIFNγ, 50 ng/mL, #315-05, #214-14, PeproTech, Hamburg, Germany), or activated to M2-polarized macrophages with interleukin-4 (mIL-4, 10 ng/mL, PeproTech, Hamburg, Germany). Cells were harvested in Qiazol buffer for RNA, and the conditioned media were collected for ELISA assays at 24 h or 48 h.

### 4.9. Immunofluorescence Staining

A series of mouse brain floating tissues of 60 μm thickness were fixed in 4% PFA. The slices were stained using a free-floating protocol. After the slices were washed 6 times with PBS for 10 min each, blocking with Blocking Solution (POLHRP-100, Zytomed Systems, Berlin, Germany) for 1 h at room temperature on a shaker was performed. Afterwards, slides were incubated overnight with 400 µL of primary antibody diluted with Antibody Diluent (ZUC025-100, Zytomed Systems, Berlin, Germany) on a shaker at 7 °C. The following antibodies were used: anti-F4/80 (1:8000 dilution, MCA497G, Bio-Rad, Feldkirchen, Germany), anti-iNOS (NOS2, 1:1000 dilution, ab3523, Abcam, Cambridge, UK), and anti-Arg1 (1:16,000 dilution, ab91279, Abcam, Cambridge, UK). The slices were then washed 3 times with PBS + 0.3% Triton X-100 for 20 min each. Each of the following steps was performed under light protection: The slices were incubated with secondary antibodies goat anti-rat 488 and goat anti-rabbit 546 (1:1000 dilution for both, A-11006 and A-11010, Invitrogen, Waltham, MA, USA) for 4 h at room temperature. Thereafter, the slices were washed with PBS + 0.3% Triton X-100, then with PBS + 1µg/mL DAPI, and then with PBS for 20 min each. To clear the slices, they were incubated in 60% glycerol and 40% dH_2_O (distilled water) with 2.5 M fructose as described in [80]. Slices were mounted on slides using the glycerol–water–fructose mixture. Slices of murine colon and liver served as positive controls for iNOS (NOS2) and Arginase 1 (Arg1), respectively, as well as for F4/80 (Supplementary Figure S6). Image acquisition was performed using a Leica SP8i confocal microscope at 408, 488, and 552 nm. For image analysis, QuPath software (version V0.4.4) was used [67]. Automatic classification of macrophages was achieved using an object classifier that was trained to detect different types of macrophages based on a random tree analysis (RTrees). 

### 4.10. Statistics, Algorithms, and Tools Used for Data Analyses

Statistics: For RT-PCR analyses, values in each group were first inspected for normality with the Kolmogorov–Smirnov test, and then the groups were compared using ANOVA, using the post-hoc test Tukey for multiple comparisons. For correlation inspection, Pearson correlation was calculated for each pair of values, and the values were represented as heatmaps. The significance level was set to 0.05, and the trend to significance was set to values of 0.1 < *p* < 0.05.

The correlation of ADAM8 and PD-L1 (CD274) in different tumor types was assessed with Timer 2.0 (http://timer.cistrome.org/, accessed on 30 October 2023) within the modules “exploration” and “gene correlation”. For the metabolomic assessment, Metaboanalyst 5.0 was used with an annotated list to investigate the pathways involved, using the HMDB numerical codes as input for each metabolite. 

## 5. Conclusions

Our results suggest that the MRSI-based noninvasive surrogate biomarker is sampling relevant changes in the GB tumor microenvironment, related both to changes in macrophage population and polarization status. Moreover, even local changes triggered by metalloproteinases can be monitored. Local changes are relevant for therapy response and have translational potential, i.e., by estimating the success rate of immunotherapy or even by modulating the cleavage of membrane-bound PD-L1 to optimize immunotherapy of GB. 

## Figures and Tables

**Table 1:** Summary of the cases included in this work and tumor volume evolution observed.

Group	Total Number of Cases	Evolution	Cases with RT-PCR Studies	Cases with Immunofluorescence Studies	Tumor Volume at Therapy Starting Point (11 p.i. *) (mm^3^)	Tumor Volume at Euthanasia (mm^3^)	Euthanasia Day
IMS-TMZ-treated responding, maximum of MRSI-based biomarker (high TRI) **	17 **	Tumor volume met criteria for “stable disease” according to RECIST [19]; adapted as described in [20] and showing TRI values > 60%	12	5	7.9 ± 3.1	54.2 ± 24.2	23 ± 1
Control (vehicle-treated, low TRI)	15 ***	Tumor volumes increased exponentially as expected, showing TRI values close to 0%	10	5	10.3 ± 8.3	74.4 ± 24.3	18 ± 3
IMS-TMZ-treated, relapsing	7	Tumors transiently responded to treatment and subsequently escaped therapy, showing clear regrowth	7	0	8.1 ± 4.1	149.7 ± 33.0	40 ± 4
IMS-TMZ-treated, unresponsive	3	Tumor volumes increased fast, and no signs of transient growth arrest were observed	3	0	13.7 ± 4.7	179.4 ± 36.1	22 ± 2

* p.i.—post-implantation. ** Please check the Section 1 for a description of TRI and MRSI-based biomarker. *** 10 IMS-TMZ-treated responding and 10 control cases have already been described in [16].

## Data Availability

Data supporting findings described in this work, such as raw MR data and prism files with expression levels, will be made available through *Dipòsit Digital de Documents* (ddd.uab.cat, accessed on 30 October 2023), as well as metafiles with data descriptions.

## Supplementary Materials

The supporting information can be downloaded at: https://www.mdpi.com/article/10.3390/ijms242417628/s1.
