# Supplementary material for: Correlation of MR-Based Metabolomics and Molecular Profiling in the Tumor Microenvironment of Temozolomide-Treated Orthotopic GL261 Glioblastoma in Mice
Source: Int J Mol Sci. 2023 Dec 18;24(24):17628. doi: 10.3390/ijms242417628 (PMC10743933; doi:10.3390/ijms242417628)
Supplement: Supplementary file 1 [file ijms-24-17628-s001.zip › ijms-2721858-supplementary.pdf]

Supporting information file

Correlation of MR-Based Metabolomics and Molecular Profiling in the tumor microenvironment of temozolomide-treated orthotopic GL261 glioblastoma in mice

Kai Zhao[1γ], Pilar Calero-Pérez[2,3 γ], Miriam H.A. Bopp[1], Vincent Möschl[4,]Axel Pagenstecher[4], Marta Mulero-Acevedo, Mario Vázquez, Carlos Barcia, Carles Arús, Christopher Nimsky[1], Jörg W. Bartsch[1], Ana Paula Candiota[3,2,5*]

<u>Supplementary Materials:</u>

MRI/MRSI Acquisition

Data were acquired in a 7T Bruker BioSpec 70/30 USR spectrometer (Bruker Biospin GmbH, Ettlingen, Germany) equipped with a mini-imaging gradient set (400 mT/m). A 72-mm inner-diameter linear volume coil was used as transmitter, and a mouse brain surface coil as a receiver for brain MRI studies. A dedicated bed was used for mouse placement, which allowed delivery of anaesthesia (isoflurane, 1.5%-2.0% in O_2 at 1 L/min). Body temperature regulation was performed with an integrated heating water circuit. The respiratory frequency was kept between 60 and 80 breaths/min and it was monitored with a pressure probe.

MRI studies

High-resolution coronal T2w images were acquired using a Rapid Acquisition with Relaxation Enhancement (RARE) sequence, with repetition time (TR)/effective echo time (TEeff) = 4200/36 ms. The acquisition parameters were as follows: repetition time (TR)/effective echo time (TEeff) = 4200/36 ms; echo train length (ETL) = 8; field of view (FOV) = 19.2 × 19.2 mm; matrix size (MTX) = 256 × 256 (75 × 75 μm/pixel); number of slices (NS) = 10; slice thickness (ST) = 0.5 mm; inter-ST = 0.1 mm; number of averages (NA) = 4; total acquisition time (TAT) = 6 min and 43 s. MRI data were acquired and processed using ParaVision 5.1 software (Bruker BioSpin GmbH, Ettlingen, Germany).

MRSI studies

MRSI was acquired with point-resolved spectroscopy (PRESS) localization sequence at 14 ms echo time (TE), and grids were positioned individually across the tumor, using as a reference T2w high-resolution MRI, as described in

previous work [3]. In general, three to four MRSI grids were enough to cover the whole tumor. The matrix size was 10 × 10 or 12 × 12. Shimming was performed individually for each MRSI grid, which were carefully placed ensuring that the volume of interest (VOI) included most of the tumor area and normal/peritumoral brain parenchyma.

Acquisition parameters for all grids were: FOV, 17.6 mm × 17.6 mm; VOI in Grids 1 and 4 was 5.5 mm × 5.5 mm × 1.0 mm. VOI in Grids 2 and 3 was 6.6 mm × 6.6 mm × 1.0 mm, with a resulting pixel size of 0.55 x 0.55 x 1.00 mm. ST, 1 mm; TR, 2500 ms; Sweep Width (SW), 4006.41 Hz; NA, 512; TAT, 21 min 30 s each grid. Water suppression was performed with Variable Power and Optimized Relaxation Delay (VAPOR), using a 300 Hz bandwidth. Linear and second order shims were automatically adjusted with Fast Automatic Shimming Technique by Mapping Along Projections (FASTMAP) in a 5.8 mm × 5.8 mm × 5.8 mm volume which contained the VOI region. Six saturation slices (ST, 10 mm; sech-shaped pulses: 1.0 ms/20250 Hz) were positioned around the VOI to minimize outer volume contamination in the signals obtained.

MRI and MRSI processing and post-processing

Tumor volume calculation: manual segmentation of T2w high-resolution horizontal images and tumor volumes were calculated using the following equation:

$$TV(mm^3) = [(AS_1 \times ST) + [AS_2 + (\dots) + AS_n) \times (ST + IT)]] \times 0.075^2$$

where TV is the tumor volume, AS is the total number of pixels in the region of interest in each MRI slice, ST is the slice thickness, IT the inter-slice thickness and 0.075^2 the individual pixel surface area in mm^2. The inter-slice volume was estimated adding the inter-slice thickness to the corresponding slice thickness in the previous equation.

MRSI data were initially pre-processed at the MR workstation with ParaVision 5.1, and then post-processed with 3D Interactive Chemical Shift Imaging (3DiCSI) software package version 1.9.17 (Courtesy of Truman Brown, PhD, Columbia University, New York) performing adjustments related to line broadening (Lorentzian filter, 4 Hz) and zero-order phase. Data was exported to ASCII format and a home-developed processing module Dynamic MRSI (DMPM), running over MatLab 2013a (The MathWorks Inc., Natick, Massachusetts) was used to align spectra within each MRSI matrix, using the choline signal as reference, 3.21 ppm). The 0 to 4.5 ppm region of each spectrum in the MRSI matrix was normalized to unit length and exported in ASCII format

for performing the PR analysis. No baseline correction was performed in these spectra. A previously described semi-supervised approach [13] which relies on Convex-NMF was used for classifying pixels into normal brain parenchyma, actively proliferating tumor and tumor responding to treatment, and for calculating nosologic maps representing the spatial response to treatment. Green color is used when the GB responding to treatment source contributes the most, blue for normal brain parenchyma, red for actively proliferating GB and black for undetermined tissue. From the biochemical viewpoint, the source extraction technique to classify MRS data assumes that in each voxel there is a mixture of heterogeneous tissues and their metabolites from which the contribution of each source can be obtained.

Inclusion criteria for mice used in this study

Control and TMZ-treated, responding groups (having both MRI and MRSI data)

1. Homogeneous tumor appearance at MRI exploration, searching for tumor volume range values (average 9.3 ± 6.1 mm^3) obtained in previous cohort studies from our group. Tumors growing towards the skull or lower part of the brain, in which MRSI signals can have poor spectral quality, were also disregarded.
2. Extreme values for Tumor Responding Index (i.e., TRI values >60% for treated tumors and close to 0% for control tumors). In addition, homogeneous TRI distribution within the tumor mass was preferred, i.e. mostly homogeneous nosological images.
3. Maintenance or reduction in the tumor size was searched for in the treated group, pointing towards clear growth arrest. The reduction was mostly in agreement with 'stable disease' according to RECIST values. The lack of further follow-up prevented us to define whether it would meet partial or complete response criteria.
4. Tumor tissue available with volume large enough (more than 20 mm^3) to obtain the amount of RNA suitable to carry out qPCR experiments (i.e. > 70 ng/μL). It is worth noting that mice discarded from this study because of inclusion criteria, were allocated to other studies performed by our group in order to minimize animal waste and maximize knowledge obtained from this particular animal model.

For relapsing and treated-unresponsive cases, only MRI data was acquired. Relapsing cases showed clear transient growth arrest (or volume decrease), followed by exponential regrowth, characterizing transient response according to our previous studies. Unresponsive cases did not show any sign of growth arrest, behaving mostly like control, untreated cases (please refer to Figure 1A of the main manuscript for a visual representation of tumor growth evolution).

Adapted RECIST criteria

Classification of adapted RECIST criteria was applied as follows: Progressive disease (PD): 20% increase with respect to the smallest tumor volume so far. Partial response (PRe): tumor decrease by 30%, taking into account the biggest volume so far. Stable disease (SDi): less than 20% increase and no more than 30% decrease in tumor volume.

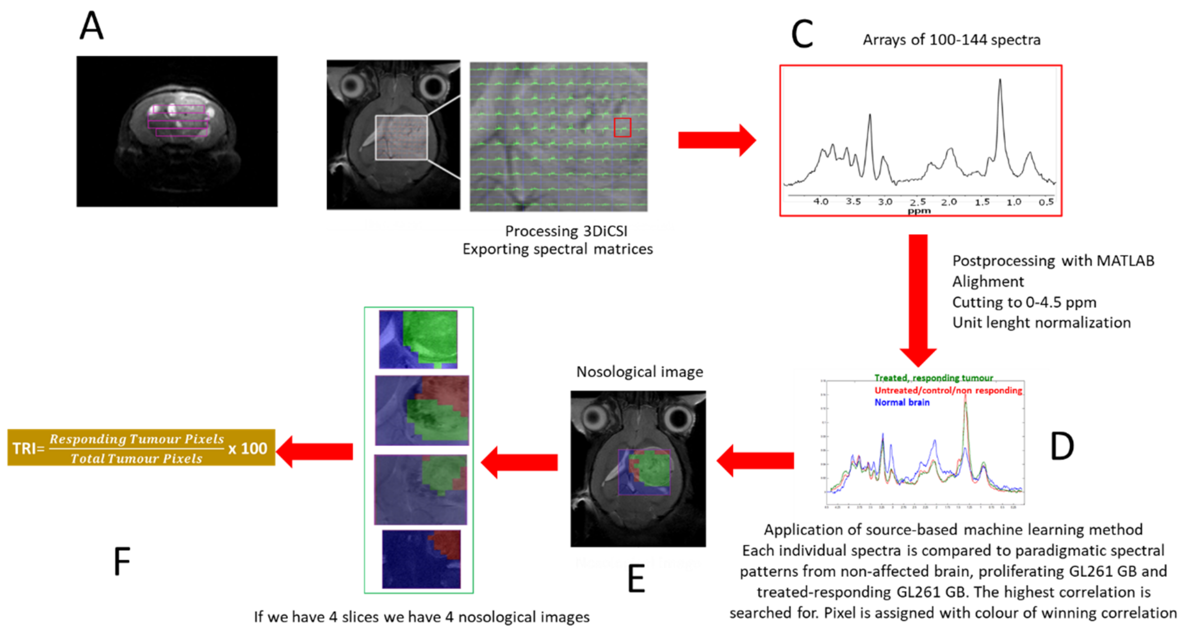

Supplementary Figure S1. Workflow for nosological imaging generation and TRI calculation. (**A**) Three to four MRSI slices positioned along the GL261 GB tumor mass (coronal view); (**B**) each one of the MRSI slices produces an array of 10x10 or 12x12 spectra (shown in axial view), which were processed with 3DiCSI software and (**C**) matrices of spectra were exported, to be further processed with MATLAB modules for alignment, spectral cutting and unit length normalization. The generated file will be the input for (**D**) analyses with the source-based machine learning developed method, in which each individual spectra is compared to previously extracted paradigmatic spectra from unaffected brain, control untreated tumors and TMZ-treated, responding tumors. The highest correlation with a given source will determine the color of a given pixel, generating (**E**) the nosological image, after every pixel has received a color (blue: unaffected brain parenchyma, red: untreated, proliferating tumor, green: TMZ-treated, responding tumor). Process is repeated for each slice (total, 3 to 4 nosological images). (**F**) Calculation of tumor responding index (TRI) from nosological images, i.e. total of pixel identified as responding divided by total tumor pixels. In order to guide sample obtention, untreated tumors had close to 0% value, and responding tumors were chosen with values above 65% (this should be accompanied by tumor growth arrest or volume decrease (see also section 4.3 of the main manuscript)

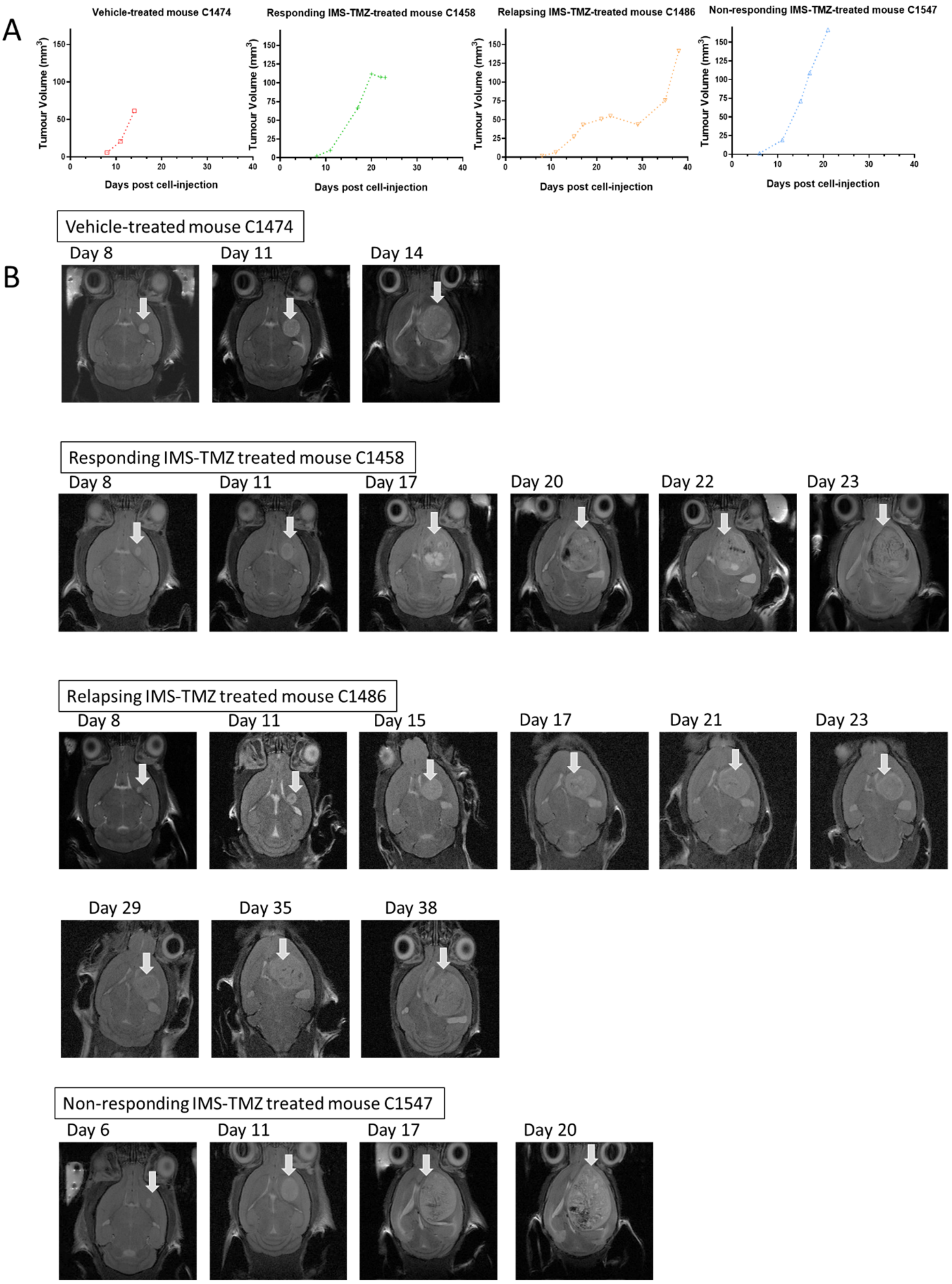

Supplementary Figure S2. A representative example of each described group (control, red line; IMS-TMZ treated and responding, green line; IMS-TMZ treated and relapsing, orange line; IMS-TMZ treated, non-responding, blue line) is shown, (**A**) tumor growth curve with values calculated from MRI data illustrated in (**B**). Arrows point to the tumor mass in each case.

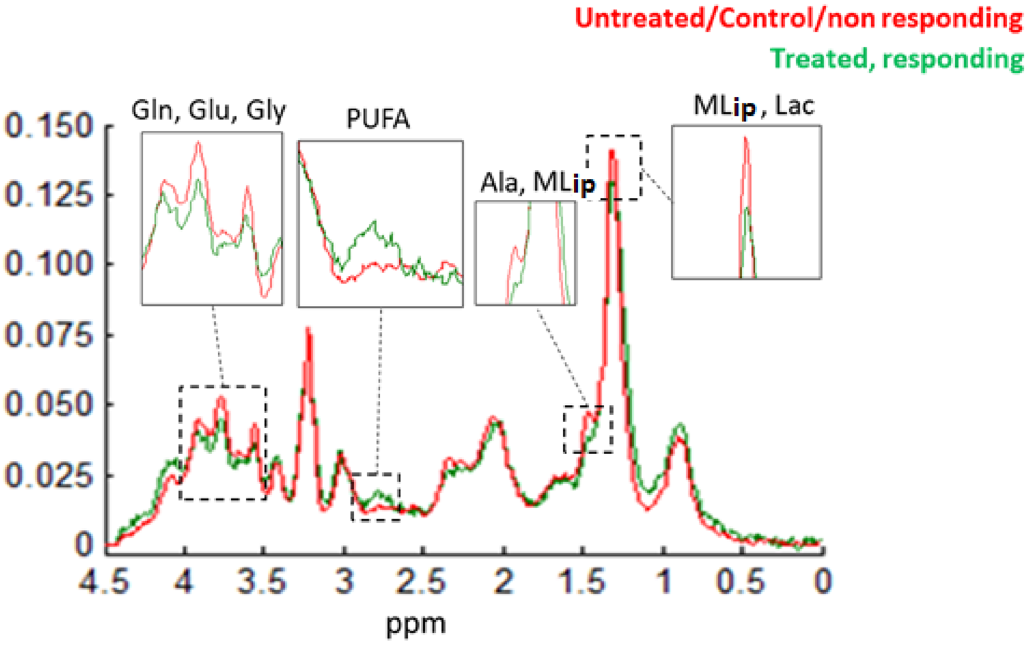

Supplementary Figure S3. Small, but consistent differences found in spectral pattern which can agree with metabolomic changes in GB cells and/or changes in macrophage phenotypes. Note that these are **not** individual spectra, but paradigmatic patterns (sources) mathematically extracted from a high number of individual labeled spectra from TMZ treated and untreated mice. Abbreviations: Gln, glutamine; Glu, glutamate; Gly, glycine; PUFA, polyunsaturated fatty acids; Ala, alanine; MLip, mobile lipids and Lac, lactate.

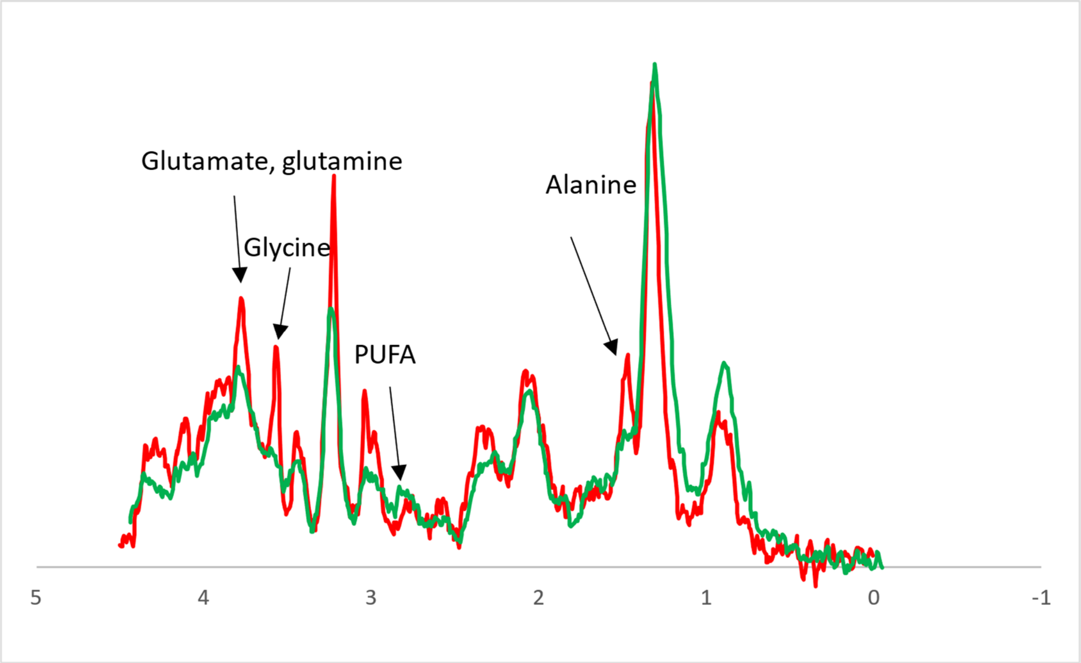

Supplementary Figure S4. Representative spectral pattern of a voxel predicted as tumor (C1474, slice 2, control mouse) and of a voxel predicted as responding (C1458, slice 2, IMS-TMZ treated mouse). Cases shown are the same on Figure 1B of the main manuscript. Abbreviations: Gln, glutamine; Glu, glutamate; Gly, glycine; Ala, alanine; ML, mobile lipids and Lac, lactate. Arrows signal the consistency of spectral pattern changes observed in mathematically extracted paradigmatic spectra (Supplementary Figure 2) and real average spectra in this figure.

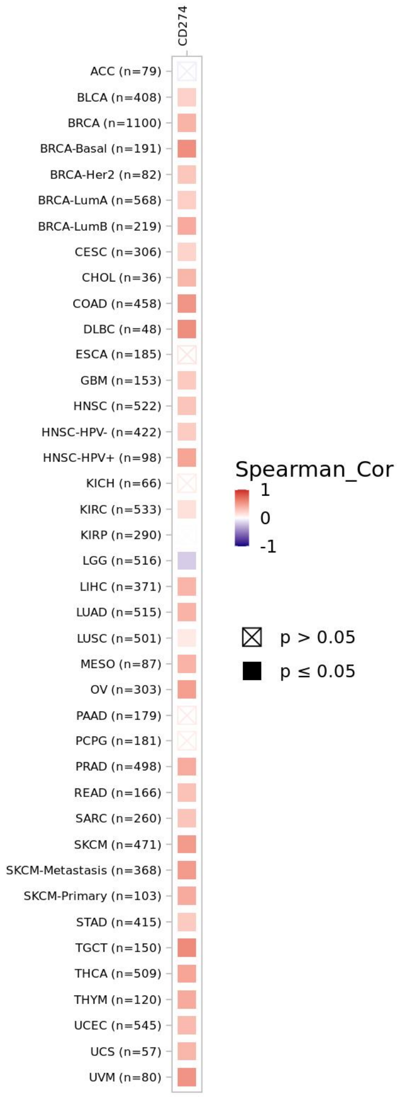

Supplementary Figure S5. Correlation between ADAM8 and CD274 (PD-L1) expression in different cancer types according to TIMER 2.0. Please see http://timer.cistrome.org/ for acronyms and information.

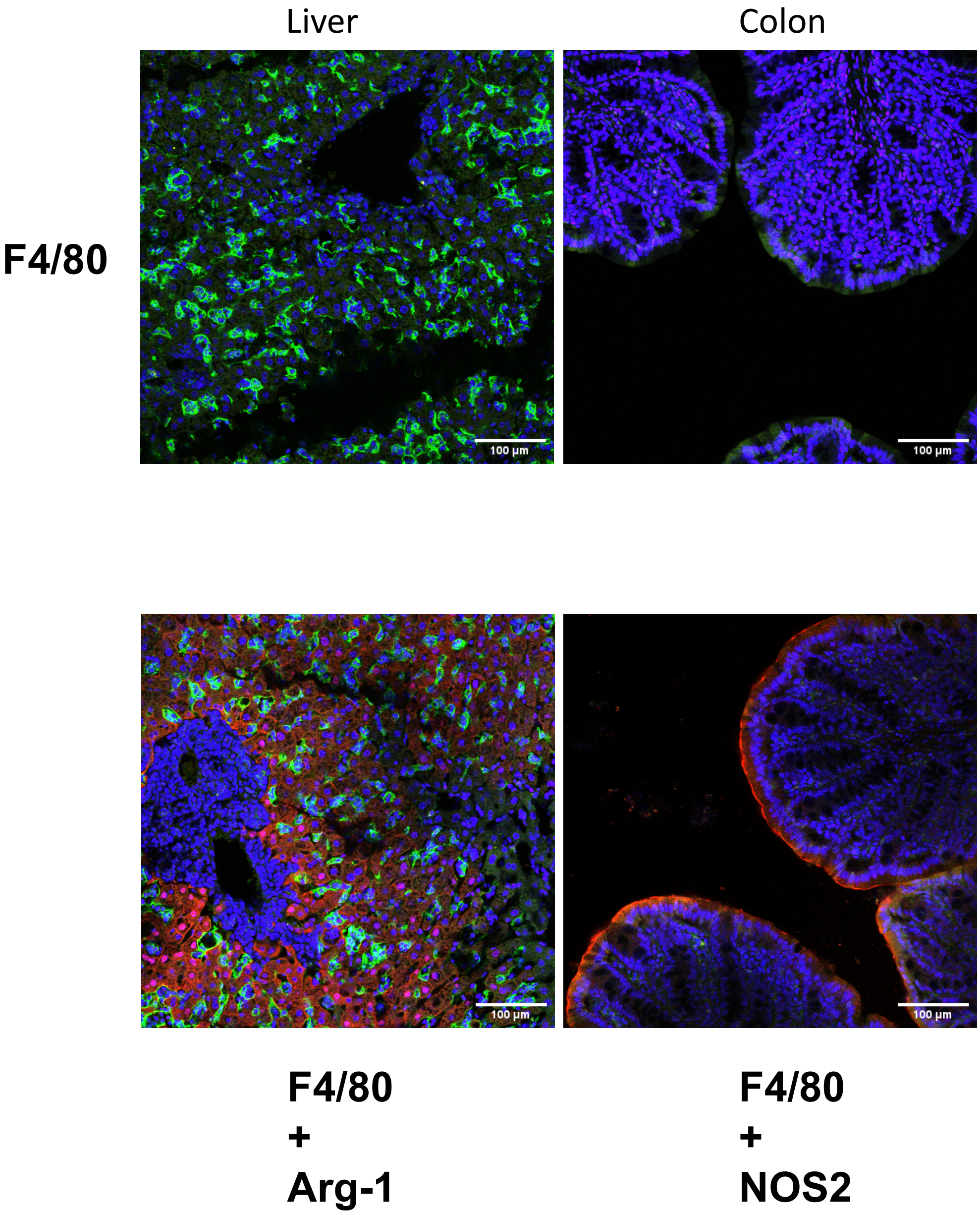

Supplementary Figure S6. Positive controls for the antibodies used to detect macrophage populations in GB slices. Here, liver tissue was used as positive control for F4/80 (liver) and for colon (weak staining in the lamina propria). Numerous F4/80 positive macrophages/Kupffer cells can be well appreciated within the liver tissue. Furthermore, liver tissue was used as positive control for Arginase-1 antibody, while colon epithelium is positive for NOS2.

Supplementary Tables:

Table S1. full list of studied animals with details on tumor volumes (mm^3) at starting point, at euthanasia time point, euthanasia day and TRI in % (see section 4.3), na = not available.

Group	Code number	Tumor volume at IMS-TMZ treatment starting point	Tumor volume at euthanasia point	Euthanasia day	TRI %
IMS-TMZ treated, responding	C1412	12.05	87.17	23	95.21
	C1445	3.74	45.99	23	60.13
	C1447	6.17	94.92	28	78.70
	C1450	5.45	29.61	24	72.83
	C1451	11.6	54.09	23	64.24
	C1456	3.63	24.5	23	71.18
	C1458	9.95	107.24	23	81.78
	C1460	9.3	59.12	23	93.57
	C1463	4.37	35.55	23	76.47
	C1473	8.69	71.96	23	75.61
	C1504	7.34	49.09	23	na
	C1505	6.41	44.48	23	na
Control, vehicle-treated	C1320	5.8	64.36	18	3.26
	C1344	4.26	29.3	17	0.00
	C1348	4.35	109.67	21	5.37
	C1457	1.12	54.86	23	6.84
	C1465	13.02	66.05	15	0.85
	C1466	1.21	72.36	23	0.00
	C1467	2.56	130.03	22	6.00
	C1471	12.55	78.08	17	35.80
	C1472	24.92	57.26	13	2.66
	C1474	20.87	61.26	14	0.00
IMS-TMZ treated, relapsing	C1380	10.09	172.72	41	na
	C1383	4.39	183.86	49	na
	C1410	15.08	96.56	36	na
	C1486	6.24	141.14	38	na
	C1489	5.77	125.52	38	na
	C1491	4.02	185.66	38	na
	C1494	10.85	142.41	42	na
IMS-TMZ treated, unresponsive	C1493	11.86	151.45	23	na
	C1496	10.16	220.13	23	na
	C1547	19.08	166.73	21	na

References: (numbering matches numbers used in the main manuscript)

[8] Arias-Ramos, N.; Ferrer-Font, L.; Lope-Piedrafita, S.; Mocioiu, V.; Julià-Sapé, M.; Pumarola, M.; Arús, C.; Candiota, A.P. Metabolomics of Therapy Response in Preclinical Glioblastoma: A Multi-Slice MRSI-Based Volumetric Analysis for Noninvasive Assessment of Temozolomide Treatment. Metabolites 2017, 7, 20.

[20] Delgado-Goñi, T.; Ortega-Martorell, S.; Ciezka, M.; Olier, I.; Candiota, A.P.; Julià-Sapé, M.; Fernández, F.; Pumarola, M.; Lisboa, P.J.; Arús, C. MRSI-Based Molecular Imaging of Therapy Response to Temozolomide in Preclinical Glioblastoma Using Source Analysis. NMR Biomed. 2016, 29, 732–743.
